# A Randomized Clinical Trial Study: Anti-Oxidant, Anti-hyperglycemic and Anti-Hyperlipidemic Effects of Olibanum Gum in Type 2 Diabetic Patients 

**Published:** 2014

**Authors:** Abbas Azadmehr, Amir Ziaee, Laleh Ghanei, Hassan Fallah Huseini, Reza Hajiaghaee, Bahareh Tavakoli-far, Gholamreza Kordafshari

**Affiliations:** a *Metabolic Disease Research Center, Department of Immunology, Qazvin University of Medical Sciences, Qazvin, Iran. *; b *Metabolic Disease Research Center, Qazvin University of Medical Sciences, Qazvin, Iran. *; c *Pharmacology and Applied Medicine, Department of Medicinal Plants Research Center, Institute of Medicinal Plants, ACECR, Karaj, Iran. *; d *Pharmacognosy and Pharmaceutic Department of Medicinal Plants Research Center, Institute of Medicinal Plants, ACECR, Karaj, Iran. *; e *Department of Pharmacology, Alborz University of Medical Sciences, Karaj, Iran. *; f *School of Traditional Medicine, Tehran University of Medical Sciences, Tehran, Iran. *

**Keywords:** Olibanum gum resin, Diabetes, Hyperglycemia, Hyperlipidemia, Patients

## Abstract

Diabetes is a common metabolic disease in the world that has many adverse effects. Olibanum gum resin (from trees of the genus Boswellia) has traditionally been used in the treatment of various diseases such as diabetes. The aim of this study was the comparison of Olibanum gum resin effect with placebo on the treatment of type 2 diabetes. Inclusion criteria was diabetic patients with fasting blood sugar (FBS) =140-200 mg/dL. This study has been designed as double-blined clinical trial on 71 patients with type 2 diabetes and the patients randomly were divided to interventional and placebo groups. The patients on standard anti-diabetic therapy (metformin) treated with Olibanum gum resin (400 mg caps) and placebo tow times per day for 12 weeks, respectively. At the end of the twelfth week, the FBS, HbA1c, Insulin, total Cholesterol (Chol), LDL, Triglyceride (TG), HDL and other parameters were measured. The Olibanum gum resin lowered the FBS, HbA1c, Insulin, Chol, LDL and TG levels significantly (p < 0.001, p < 0.001, p <0.001, p = 0.003, p < 0.001 and p < 0.001, respectively) without any significant effects on the other blood lipid levels and liver/kidney function tests (p > 0.05) compared with the placebo at the endpoint. Moreover, this plant showed anti-oxidant effect and also no adverse effects were reported. The results suggest that Olibanum gum resin could be used as a safe anti-oxidant, anti-hyperglycemic and anti-hyperlipidemic agent for type 2 diabetic patients.

## Introduction

Diabet mellitus type 2 is the most common metabolic disease in the world (1). Several anti-hyperglycemic and anti-hyperlipidemic drugs are used for treatment of type 2 Diabetes mellitus (2). Using of these drugs has limited efficacies and important adverse effects. Therefore, recognition of new agents as plant medicines with more efficacious and safety are needed (3). Apart from conventional anti diabetic therapy; medicinal plants, complementary and alternative medicine therapies have beneficial effects and improve glucose homeostasis in diabetic patients (4-6). Olibanum gum resin, as a medicinal plant, is traditionally used in India to treat various diseases including inflammatory ailment, arthritis, cardiac disorder and pain (7). Several studies showed that boswellic acids are the major constituents on the Olibanum gum resin and there have anti-inflammatory, anti-cancerouse and anti-ulcerous activities (8-11). Moreover, *in-vivo *and *in-vitro *studies show that boswellic acids were found to inhibit the synthesis of pro-inflammatory enzyme and 5-lipoxygenase such as leukotrine B4 (LTB4), which cause inflammatory reactions, bronchocostriction, chemotaxis, and increased vascular permeability (12-14). In addition, other study indicated that pure compound from *Boswellia serrata *powder exhibits anti-inflammatory activity in human peripheral blood mononuclear cells (PBMCs) and mouse macrophages through inhibition of tumor necrosis factor alpha (TNF-α), interleukin -1beta (IL-1β) and Nitric oxide (NO) inflammatory mediators (15). On the other hands, the clinical trials indicate that gum-resin of *Boswellia serrata *extract have non-toxic nature (16) and improve symptoms in patients with inflammatory disorders such as rheumatoid arthritis and osteoarthritis (17-19). Other study showed that Olibanum gum has anti-hyperglycemic and anti-hyperlipidemic effects in streptozotocin induced diabetic rats (20, 21). The new study showed that Boswellia serrata has the protective effect against long-term diabetic complications (22).Furthermore, Shehata *el al. *indicated that the gum resin of Boswellia serrata prevent islet destruction and consequent hyperglycemia in an animal model of diabetes probably by inhibition of the production/action of cytokines related to induction of islet inflammation in an autoimmune process (23). Therefore, in this study we investigated the anti-hyperglycemic and anti-hyperlipidemic effects of Olibanum gum resin in type 2 diabetic patients.

## Experimental

*Material and methods*

Olibanum gum and preparation of the Olibanum gum resin. The Olibanum gum resin with certified botanical origin, *Boswellia serata *(India), was purchased from herbal market. It was ground in to powder. The resin powder as the drug and toast powder as the placebo were separately filled into oral gelatin capsules with identical appearance by using a hand-operated capsule-filling machine (Scientific Instruments and Technology Corporation). The Olibanum gum resin capsules contained 400 mg of gum olibanum resin powder. Toast powder was chosen as the placebo, because its appearance was relatively similar to the *Gum olibanom *resin powder.

*Phytochemical assay*

Thin layer chromatocheraphy (TLC) was used for recognizing chemical components of resin. A variety of indicators including vanillin sulfuric acid; ferric chloride and natural product polyethylene glycol were used in this assay. The indicators were sprayed on prepared thin layers of fractions and were observed at 260 and 280 nm wavelengths under UV light.

*Patients*

**Figure 1:**
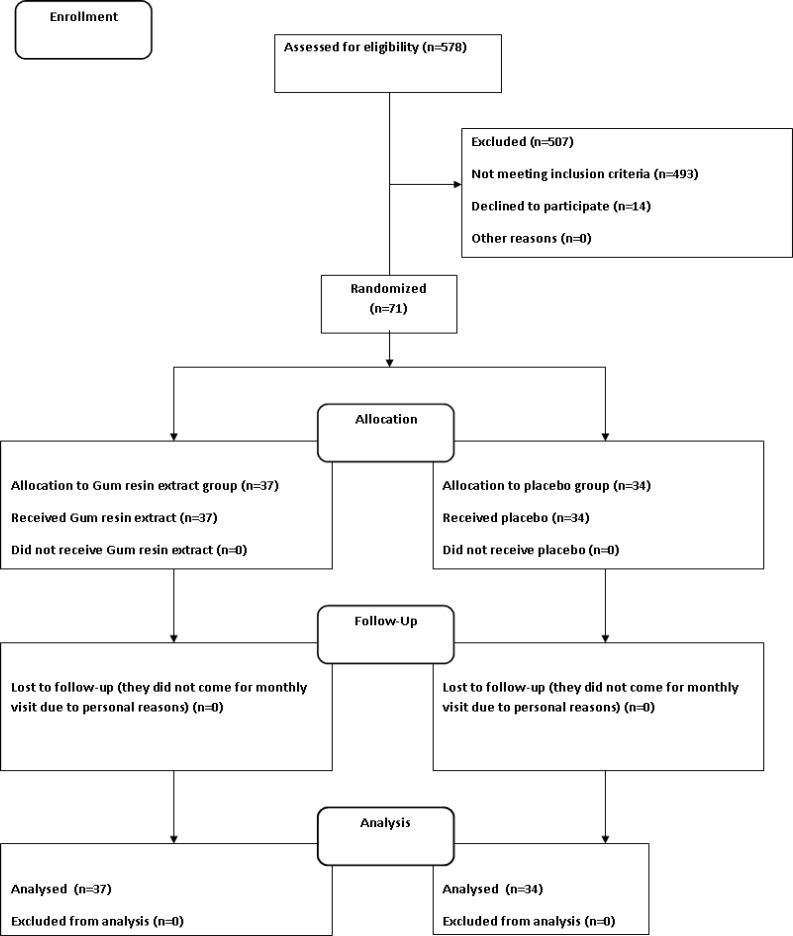
The consort flowchart describing the progress of the patients through the trial

Seventy-one Iranian male and female type 2 diabetic patients (aged 18 to 65 years) registered at the Diabetic Clinic registry of Qazvin Hospital Qazvin Iran, who were eligible according to the inclusion and exclusion criteria participated in this study. The CONSORT flowchart describing the progress of the patients through the trial is shown in Figure 1. In addition, the demographic data of the subjects who finished the trial are given in Table 1. The patients were visited by investigators and informed about the rationale and main aims of the study. Written informed consent was obtained from the patients. The medical ethics committee of the Qazvin University of Medical Sciences approved the protocol. All the patients who participated had confirmed diabetes type 2 according to ADA (24) criteria. Inclusion criteria were diabetic patients with FBS=140-200 mg/dL and HbA1c 7 to 9%. Exclusion criteria were pregnant and nursing women; people under 18 years old and above 65 years old; cases of intensive diabetic; using drugs which affect blood glucose like edible and positional steroid; diuretics drugs. In addition, the trial was registered in the Iranian Registering of Clinical trials (IRCT) with the number IRCT201112078318N1.

*Patients groups*

All 71 patients were randomly assigned to groups including 37 and 34 patients in test and placebo*. *The Olibanum gum resin and placebo capsules were randomly given to the patients. The patients and the investigators who carried out the study were unaware of treatment groups and type of medication. The patients in one group took 400 mg Olibanum gum resin capsules and patients in other group took 400 mg placebo capsules two times a day after meal. The conventional oral anti-hyperglycemic agent treatments were continued in two groups. 

*Protocol*

The Body mass index (BMI), Body weight, Waist circumference levels, fasting blood sugar (FBS), Glycosylated hemoglobin (HbA1c), Triglyceride (TG), Cholesterol (Chol), Insulin (Ins), Low density lipoprotein (LDL), High density lipoprotein (HDL), Alkaline phosphatase (ALP), Alanine aminotransferase (ALT), Aspartate aminotrasferase (AST), Urea (Ur) and Creatinine (Cr) levels were determined at the baseline and after 12 weeks of the study in both groups. Blood samples were drawn after an overnight (12 h) fasting and send to hospital central laboratory. All the blood sample parameters were measured by using commercially available kits in hospital central laboratory.

*Statistical analysis*

The software SPSS 16 used for statistical analyses. The results were presented as the mean ± standard deviation (SD). The paired t-test was used to compare all blood parameters between pre- and post-intervention. p-values less than 0.05 were considered significant. 

## Results and Discussion

Olibanum gum resin has traditionally been used in the treatment of various diseases such as inflammatory ailment, arthritis, cardiac disorder and pain (7). In this study, we used Olibanum gum resin for treatment of type 2 diabetes patients. The two groups of study were matched in regard to demographic data including age, gender, duration of diabetes, and body mass index which are shown in Table 1.

**Table 1:** The demographic data of the subjects who finished the trail. The data are given as mean ± SD.

**Parameters **	**Groups**	
	***Gum olibanum *** **resin powder (N=37) **	**Placebo (N=34) **
Age (year)	51.4 ± 12.2	50.7 ± 14.4
Gender	23 male, 14 female	19 male, 15 female
Duration of Type 2 diabetes (years)	5.8 ± 4.7	5.6 ± 3.9
Body mass index (kg/m^2^)	29.2 ± 5.8	28.3 ± 5.4

 The baseline blood levels of all parameter were not significantly (P > 0.05) different between the two groups prior to the study. Moreover, phytochemical assay by thin layer chromatography showed main components; including phenyl propanoids, Terpenoids, phenolic compounds and flavonoids were presented in the resin (Table 2). 

**Table 2:** Phytochemical results of the *Gum olibanum *resin

**Result **	**Reagents**	**Solvent systems**	**Compounds**
+	Vanillin sulfuric acid	*n*-hexane: ethyl-acetate (7.2:2.8)	Terpenoids and phenylpropanoids
+	Ferric chloride	Chloroform: ethyl-acetate: formic acid (5:4:1)	Phenolic compounds
+	Natural product reagent	Ethyl acetate: methanol: water (100:13.5:10)	Flavonoides

On the other hand, diabetes is a common metabolic disease in the world that has many adverse effects. Previously study showed that *Boswellia serrata *resin of Olibanum gum resin significantly decreased blood-glucose level on non-insulin dependent diabetes mellitus in streptozocin induced diabetic rat model (21, 24, 25). In addition, other investigations indicated hypoglycemic and other related effects of *Boswellia glabra *in alloxan-induced diabetic rats (23, 25- 28). Our results in this study showed that the Olibanum gum resin lowered the Glucose, HbA1c, Insulin, total Cholesterol, LDL and TG levels significantly (P < 0.001, P < 0.001, P < 0.001, P = 0.003, P < 0.001 and P < 0.001, respectively) without any significant effects on other parameter levels (P > 0.05) compared with the placebo group at the endpoint (Table 3). 

**Table 3:** The participant data in the *Gum olibanum* resin powder (1) group (n=37) and the placebo (2) group (n=34) at baseline and after 12 weeks of the trial.

**Parameters**	**Baseline mean (SD)**		**p-value**	**Endpoint mean (SD)**		**p-value**	**Percent change Endpoint compared to baseline**	
FBS (mg/dL)	1	156.8 (10.5)	0.739	1	129.2 (9.1)	<0.001*	1	17.6 ↓
	2	156.1 (9.2)		2	153.9 (7.5)		2	1.4 ↓
HbA1c (%)	1	7.26 ( 0.47)	0.203	1	6.9 (0.38)	<0.001*	1	4.95 ↓
	2	7.42 ( 0.44)		2	7.41 (0.2)		2	0.13 ↓
Insulin (UI/L)	1	12.3 ( 6.3)	0.272	1	9.26 (2.7)	<0.001*	1	24.7 ↓
	2	10.84 ( 2.78)		2	10.1 (1.95)		2	6.8 ↓
Chol (mg/dL)	1	173.2 (27.1)	0.878	1	162.3 (27.5)	0.003*	1	6.3 ↓
	2	172.4 ( 42.5)		2	175.7 ( 29.1)		2	1.92 ↑
LDL (mg/dL)	1	101.7 (24.8)	0.6	1	88.4 (23.3)	<0.001*	1	13.1 ↓
	2	98.6 (25.02)		2	97.3 (23.1)		2	1.4 ↓
TG (mg/dL)	1	176.1 (44.5)	0.246	1	164.6 ( 38.4)	<0.001*	1	6.53 ↓
	2	171.1 ( 36.8)		2	172.5 ( 33.3)		2	0.81 ↑

P< 0.05 was considered as statistically significant.

**Figure 2:**
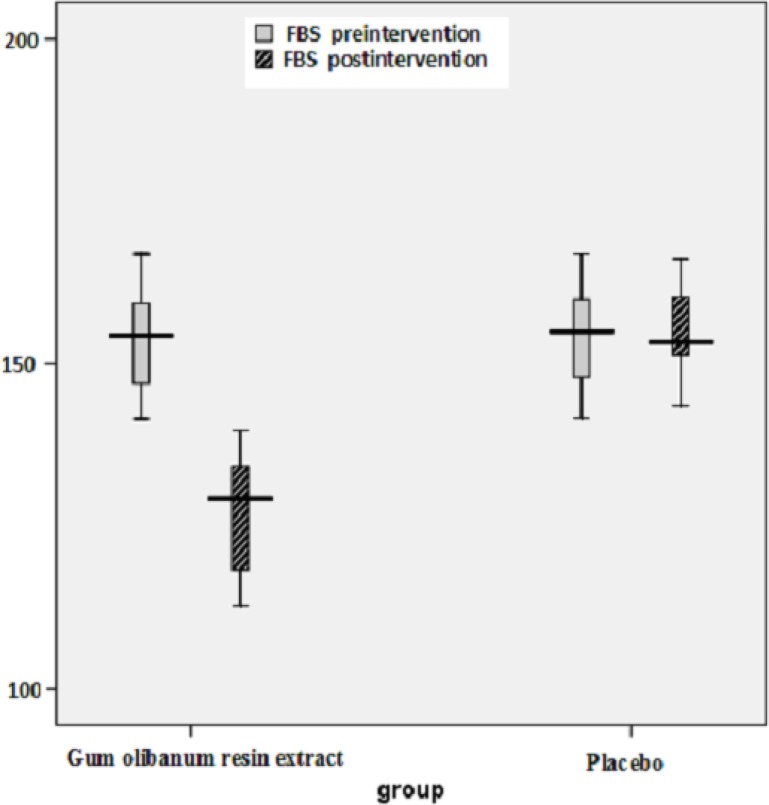
Box plot of decreases (before intervention – after intervention) in the fasting blood sugar (FBS) levels (mg/dL) of the Olibanum gum resin and placebo groups.

**Figure 3:**
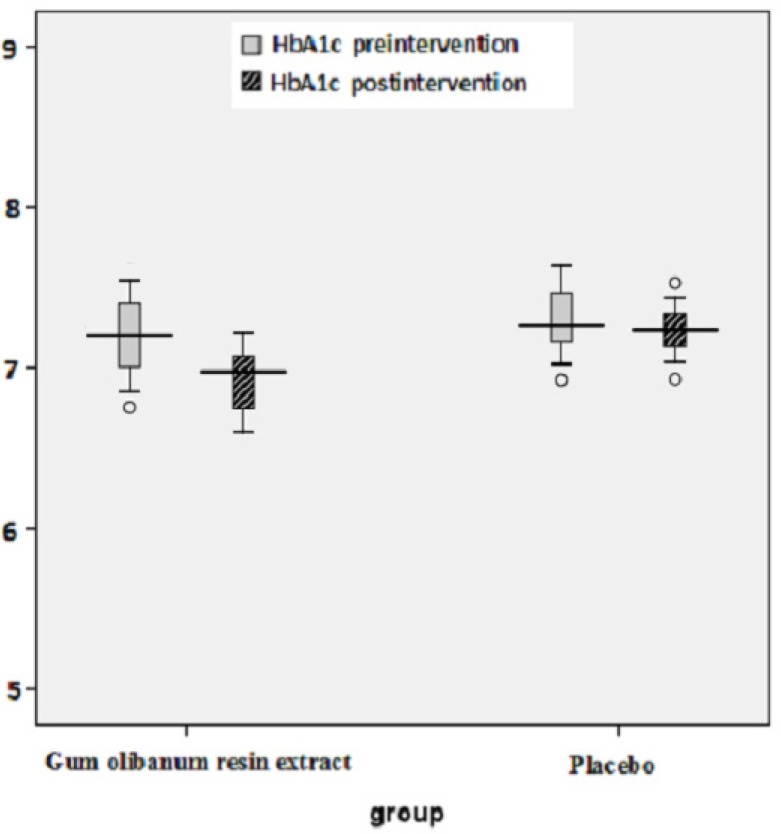
Box plot of decreases (before intervention – after intervention) in the blood glycosylated hemoglobin (HbA1c) levels (percent) of the Olibanum gum resin and placebo groups.

**Figure 4:**
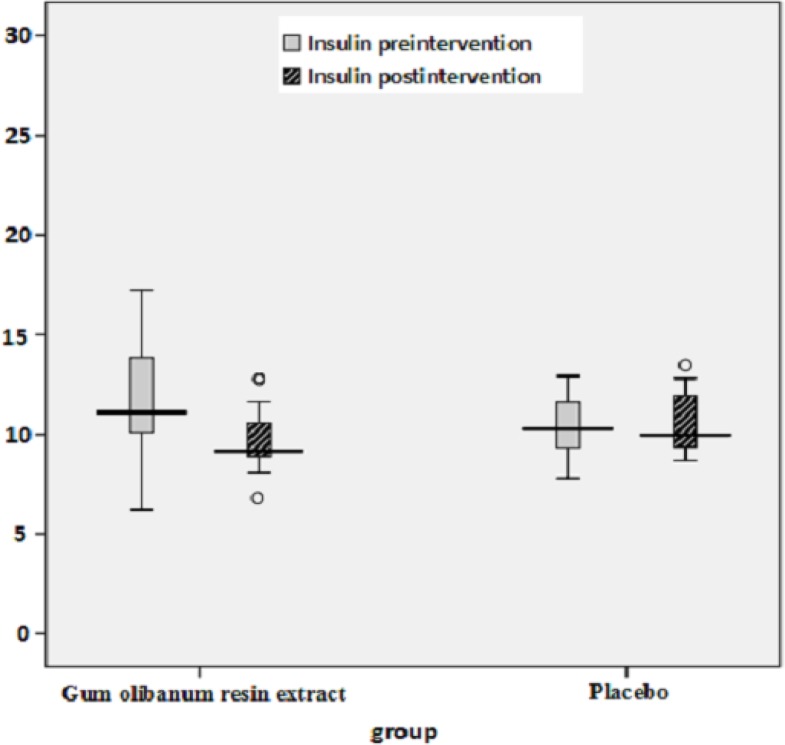
Box plot of decreases (before intervention – after intervention) in the blood insulin hormone levels (UI/L) of the Olibanum gum resin and placebo groups

**Figure 5:**
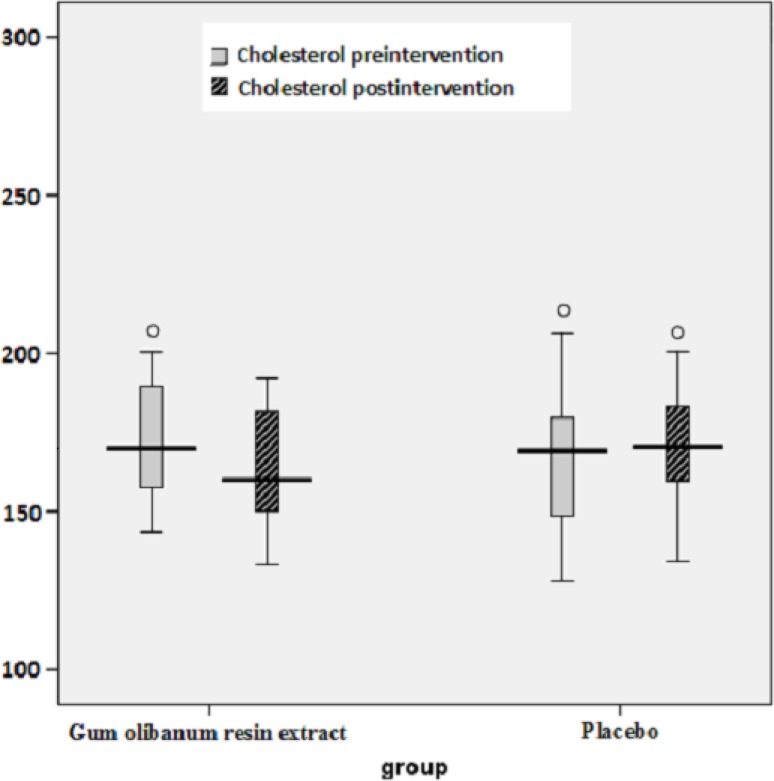
Box plot of decreases (before intervention – after intervention) in the blood total cholesterol (Chol) levels (mg/ dL) of the Olibanum gum resin and placebo groups

**Figure 6:**
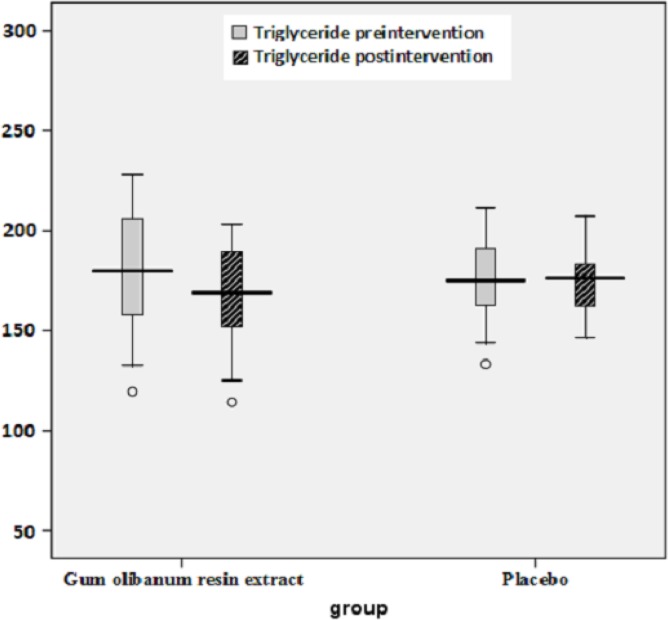
Box plot of decreases (before intervention – after intervention) in the blood triglyceride (TG) levels (mg/dL) of the Olibanum gum resin and placebo groups

**Figure 7:**
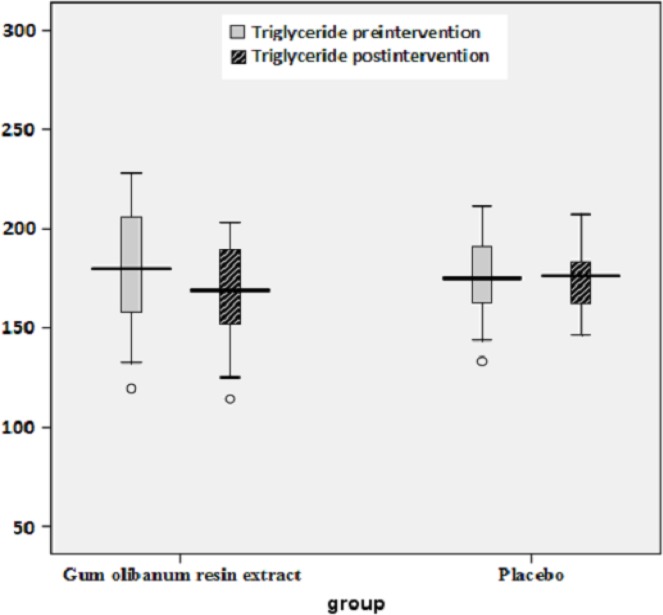
Box plot of decreases (before intervention – after intervention) in the blood LDL levels (mg/dL) of the Olibanum gum resin and placebo groups

The box plots of decreases (before intervention- after intervention) in the FBS, HbA1c, Insulin, total Cholesterol and TG levels of the Olibanum gum resin and placebo groups are shown in Figures 2-7. On the other hand, one of the best criteria for controlling of diabetes is HbA1c that shows the average of blood glucose in 2-3 past months. The result in this study showed that, using of Olibanum gum resin significantly lowered the Glucose and HbA1c in the test group comparing with placebo group. Moreover, our finding indicated that serum insulin level in the Olibanum gum resin group were significantly lowered comparing with placebo group. The hypoglycemic effect in the test group may be due to improving insulin sensitivity or other glucose metabolism mechanisms by this plant, however further studies is needed to confirm its effect. Therefore, this plant could be used as anti-hyperglycemic agent in diabetic patients. Although good glucose control is important in preventing the development of diabetes related complications, especially microvascular complications, but control of other factors such as lipids profile and blood pressure are needed in diabetic patients. Several investigations in rat’s model showed that *Boswellia serrata *extract of Olibanum gum resin significantly decreased total Cholesterol and has potential hypolipidemic and hepatoprotective activity (21, 29). In addition, our finding indicated that, using of Olibanum gum resin significantly lowered total Cholesterol and TG in the test group comparing with placebo group. So, this complementary medicinal plant could be used as anti-hyperlipidemic agent in diabetic patient. Our results in this clinical trial study suggest that Olibanum gum resin improves glycemic control and lowers the blood levels of Glucose, HbA1c, Insulin, total Cholesterol and TG; however this plant did not have either any effect on the other blood lipid parameters or any other adverse effects. In summary, considering the results of the present and previous trials and safety of Olibanum gum resin, this plant could be used as a safe anti-hyperglycemic and anti-hyperlipidemic agent for type 2 diabetic patients. However, Further and larger clinical trials seem necessary.
